# Nonlinear effects of pandemic uncertainty on depression, pandemic preventive behavior intentions, and positive life attitudes: Moderating effects of high and low uncertainty grouping

**DOI:** 10.3389/fpubh.2023.1136152

**Published:** 2023-02-23

**Authors:** Zeyu Liu, Yun Liu, Ang Li, Tingshao Zhu

**Affiliations:** ^1^Institute of Psychology, Chinese Academy of Sciences, Beijing, China; ^2^Department of Psychology, University of Chinese Academy of Sciences, Beijing, China; ^3^Dalian Vocational and Technical College, Dalian, China; ^4^Department of Psychology, Beijing Forestry University, Beijing, China

**Keywords:** normalized era of COVID-19, pandemic uncertainty, depression, positive life attitudes, preventive behavior intentions, nonlinear effects, orderly state of life, moderating effects

## Abstract

**Backgrounds:**

COVID-19 is difficult to end in a short time and people are still facing huge uncertainties. Since people's lives are gradually returning to normal, the sense of control and intolerance of uncertainty, which were mainly focused by past studies, are not specific to COVID-19 and will be more influenced by some factors unrelated to the pandemic. Therefore, they may be difficult to accurately reflect the individuals' perceptions of uncertainty. Besides, past research just after the outbreak mainly investigated people in high levels of uncertainty, we don't know the impact of uncertainties on individuals' psychological states when people gradually recovered their sense of control. To solve these problems, we proposed the concept of “pandemic uncertainty” and investigated its impact on people's daily lives.

**Methods:**

During October 20, 2021 to October 22, 2021, this study obtained data about uncertainty, depression, positive attitude, pandemic preventive behavior intentions, personality, and social support from 530 subjects using convenient sampling. The subjects were all college students from the Dalian University of Technology and Dalian Vocational and Technical College. According to the distribution of uncertainty, we divided the dataset into high and low groups. Subsequently, by using uncertainty as the independent variable, the grouping variable as the moderating variable, and other variables as the control variables, the moderating effects were analyzed for depression, positive attitude, and pandemic preventive behavior intentions, respectively.

**Results:**

The results showed that the grouping variable significantly moderate the influence of uncertainty on positive attitude and pandemic preventive behavior intentions but had no significant effect on depression. Simple slope analysis revealed that high grouping uncertainty significantly and positively predicted positive attitude and pandemic preventive behavior intentions, while low grouping effects were not significant.

**Conclusion:**

These results reveal a nonlinear effect of pandemic uncertainty on the pandemic preventive behavior intentions and positive life attitudes and enlighten us about the nonlinear relationship of psychological characteristics during a pandemic.

## 1. Introduction

Since the outbreak of COVID-19, the pandemic has never ended with the continuous variation of the virus. Nevertheless, most countries have long found a way to coexist with this pandemic, and their economies and societies are gradually returning to stable operation, instead of being stagnant as they were. Although people are used to living with a pandemic, the pandemic is like a sword of Damocles hanging over people's heads, and their lives are in great uncertainty (1–3). This uncertainty includes two aspects: (a) the uncertainty of the spread of the pandemic and of being infected; and (b) the uncertainty of the future development of the pandemic. As COVID-19 is difficult to eradicate in a short time, it is important to prevent COVID-19 from interfering with people's normal life (4). And exploring the impact of this uncertainty on psychological outcomes (like people's mental health, positive attitude toward life, and pandemic preventive behavior intentions) will help people to restore a healthy and orderly state of life.

Although previous studies have focused on this uncertainty, few studies have directly discussed this concept; they have focused more on similar concepts like a sense of control and intolerance of uncertainty. The sense of control is used to measure the extent to which individuals think they can influence events and situations in their lives (5, 6). People with higher sense of control usually think that they can decide what happens in their lives, while those with lower sense of control think that they can't decide anything (7). Besides, intolerance of uncertainty is used to measure an individual's ability to tolerate negative uncertainty (8, 9). People with higher levels of intolerance need to ensure the predictability of the future and tend to avoid unexpected events (10). During the pandemic period, a great deal of work has been done to explore the association of sense of control and intolerance of uncertainty with mental states. For example, (11) found that individuals with a lower sense of control had a greater psychological burden in pandemic-controlled areas; (12) found that teachers with lower sense of control were more strongly affected by acute stress symptoms (depression, anxiety, fear, etc.) triggered by the pandemic; A large number of studies also found that intolerance of uncertainty is a significant predictor of depression and anxiety (9, 13, 14).

However, in the “normalized era of pandemic”, it may not be appropriate in continuing to adopt the concepts of sense of control and tolerance of uncertainty. Firstly, they are not specific to COVID-19. Most studies use general scales to measure, for example, (15) used the Sense of Control Scale (16), which asks individuals about the degree of controllability and predictability of important areas of their lives (9); used the Uncertainty Tolerance Scale (17), which is used to measure individuals' cognitive, emotional, and behavioral responses of individuals to uncertain situations. The results pay more attention to the sense of control and uncertainty tolerance in one's daily life rather than to the pandemic situation. At the beginning of the outbreak, people's daily lives was greatly affected by containment and isolation measures, and the out-of-control and uncertainty mainly came from the pandemic (18, 19); whereas, at present, people's lives are gradually returning to normal, and their sense of control and uncertainty will be more influenced by some factors unrelated to the pandemic (such as factors related to individuals' work, education, or children's development), which makes it difficult to accurately reflect individuals' perceptions of the spread of the pandemic and the uncertainty of their own infection. Secondly, they are more concerned with the current state of individuals, which cannot reflect their uncertainty about the future development of the pandemic. These reasons may lead to differences between the two concepts and the concept of epidemic specificity in their effects on epidemy-related behaviors and psychological states. For example, (20) found perceptions of COVID-19 uncertainty were not associated with vaccine intentions, but tolerance of uncertainty was significantly negatively correlated with vaccine intentions (21) broke down the uncertainty and found, it was the uncertainty from various information about viruses and outbreaks rather than other uncertainties, that had a significant predictive effect on people's acute stress disorder.

Considering the reasons and the evidence above, it is urgent to find a new concept that can accurately reflect people's uncertainty about the epidemic, which will help us to investigate the impact of the epidemic more accurately on the public's psychological state and formulate appropriate intervention measures to avoid wasting strained public resources. Therefore, we define “uncertainty about the pandemic” (hereafter uncertainty) to describe individuals' views on the uncertainty of the future development of the pandemic and the uncertainty of its spread of the pandemic and infection. Individuals with high uncertainty believe that there is a higher risk of pandemic spread and infection, and they are pessimistic about the future development of the pandemic. For people with a low level of uncertainty, the situation is just the opposite.

Another question is how to measure the impact of this uncertainty on people's normal lives from a psychological perspective. A direct measure is people's positive attitude toward life in the face of the pandemic. Studies have shown that a positive attitude to life can effectively guarantee the quality of people's daily work (22), ensure people's social communication and interpersonal relationship (23), and alleviate the pain and negative impact of COVID-19 (24, 25). In addition, mental health is a vital part of a healthy life (19). In the first year of the outbreak of COVID-19, depression, anxiety, suicidal tendencies, and loneliness were on the rise all over the world (18, 19, 26). Although in the normalized era of pandemic, this trend has been eased to a certain extent (1, 27). However, it is still necessary to explore the impact of uncertainty on mental health to ensure people's normal life. Finally, with COVID-19 not yet eradicated, preventive behaviors such as wearing masks, disinfecting regularly, and reducing outings are gradually becoming part of people's daily routines (28). Therefore, exploring the influence of uncertainty on pandemic preventive behavior intentions not only guarantees people's daily lives but also has far-reaching significance to curb the spread of the pandemic situation.

Since uncertainty is a new concept, it is necessary to figure out how uncertainty affect people's positive attitude toward life, mental health, and behavioral tendencies toward pandemic prevention. Evidence from a large number of related concepts shows that people's sense of control and tolerance of uncertainty during a pandemic is an important predictor of mental health problems such as depression, anxiety, and suicidal tendencies (9, 12, 14, 18, 26). In turn, losing control can lead to the individual's strong desire to regain a sense of control, which will lead to impulse consumption (29), addictive social media use (15), and more frequent protective behaviors (30). However, it should be noted that after the outbreak of the epidemic, people's sense of control was generally reduced (31), which indicated that people were in a special state of stress (31). Although many meaningful results were obtained in this period, the research explored the relationship between people's mental state and sense of control under stress. In the normalized era of pandemic, although some people were still at a low level of control, a considerable number of people gradually recovered their sense of control. Detecting differences in the influence of uncertainty across populations was necessary, which will help us implement the interventions more accurately to avoid wasting strained public resources. Therefore, instead of investigating the average effect of the population, we should investigate whether the previous conclusions hold for people with low sense of control, and how uncertainty affects people with high sense of control.

To sum up, this study suggests that the uncertainty of pandemic situation can be used to describe the uncertainty of individual's future development of pandemic situation, as well as the uncertainty of pandemic spread and self-infection. On this basis, this study further explores the impact of epidemiological uncertainty on people's positive attitude toward life, depression levels and pandemic preventive behavior intentions. This study **hypothesized** that the high and low grouping of uncertainty moderates the effect of uncertainty itself on psychological states. When the level of pandemic uncertainty is high, people's pandemic preventive behavior intentions and depression levels increase and positive life attitudes diminish as uncertainty increases; When the level of uncertainty is low, this effect will be reduced or even disappear.

## 2. Materials and methods

### 2.1. Procedure and participants

In this study, data were collected through an online questionnaire, and the data collection time was from October 20, 2021, to October 22, 2021. Convenient sampling was performed, and the questionnaire was distributed *via* social media. Using G-power, we calculated that the minimum sample size was 487 when the significance level was 0.05, the statistical power was 0.8, and the effect size was 0.04. The subjects of the study are college students from the Dalian University of Technology and Dalian Vocational and Technical College and were filtered by polygraph questions and response times. Finally, 530 valid data were collected. The demographic information of the subjects is shown in Table 1.

**Table 1:** Demographics information.

**Demographic**	***N***	**%**
**Gender**		
Male	314	59.25
Female	216	40.75
**Region**		
East	428	80.75
Middle	60	11.32
West	42	7.92
**Household registration**		
City	172	32.45
Town	108	20.38
Rural	250	47.17
**Healthy status**		
Very well	374	70.57
Good	100	18.87
General	48	9.06
Not very well	6	1.13
Bad	2	0.38
**The severity level of COVID-19 in your hometown**		
1: Not severe	407	76.79
2: Less severe	63	63
3: Usually severe	44	8.3
4: More severe	6	1.13
5: Very severe	10	1.89

### 2.2. Measures

#### 2.2.1. Uncertainty sense toward COVID-19

During the pandemic, a self-made scale was used to measure residents' uncertainty. The scale contains six questions (Table 2) that asks subjects about their perceptions of the spread of the pandemic and their own infection, as well as their perceptions of the future development of the pandemic. The Likert scale with 5 points was adopted for all questions (from 1 strongly disagreed to 5 strongly agreed). The higher the subjects' average score, the higher their perception of the risk of pandemic spread and infection, and the higher their pessimistic perception of the future development of the pandemic. In this study, the Cronbach's α score for this scale was 0.93.

**Table 2:** Survey on uncertainty sense, positivity attitude and preventive behavioral intentions.

**Factors**	**Items (1 strongly disagree to 5 strongly agree)**
*Uncertainty sense toward COVID-19*	I always compare myself with the symptoms of pneumonia.
	I feel like I'm going to break down at any moment.
	I feel overwhelmed by this epidemic.
	I keep thinking that the epidemic will get out of control.
	I am very pessimistic about the future development of the epidemic.
	I feel very dangerous when I see strangers coming toward me
*Positive attitude*	I have been exercising during the pandemic.
	During the pandemic, I'm focused on doing what I've always wanted to do.
	During the pandemic, I develop my interest.
*Preventive behavioral intentions*	During the pandemic, I manage to cut down on going out.
	During the pandemic, I wear a mask when going out.
	During the pandemic, I advise my family to wear masks.
	During the pandemic, I wash my hands and disinfect frequently.

**Figure 1:**
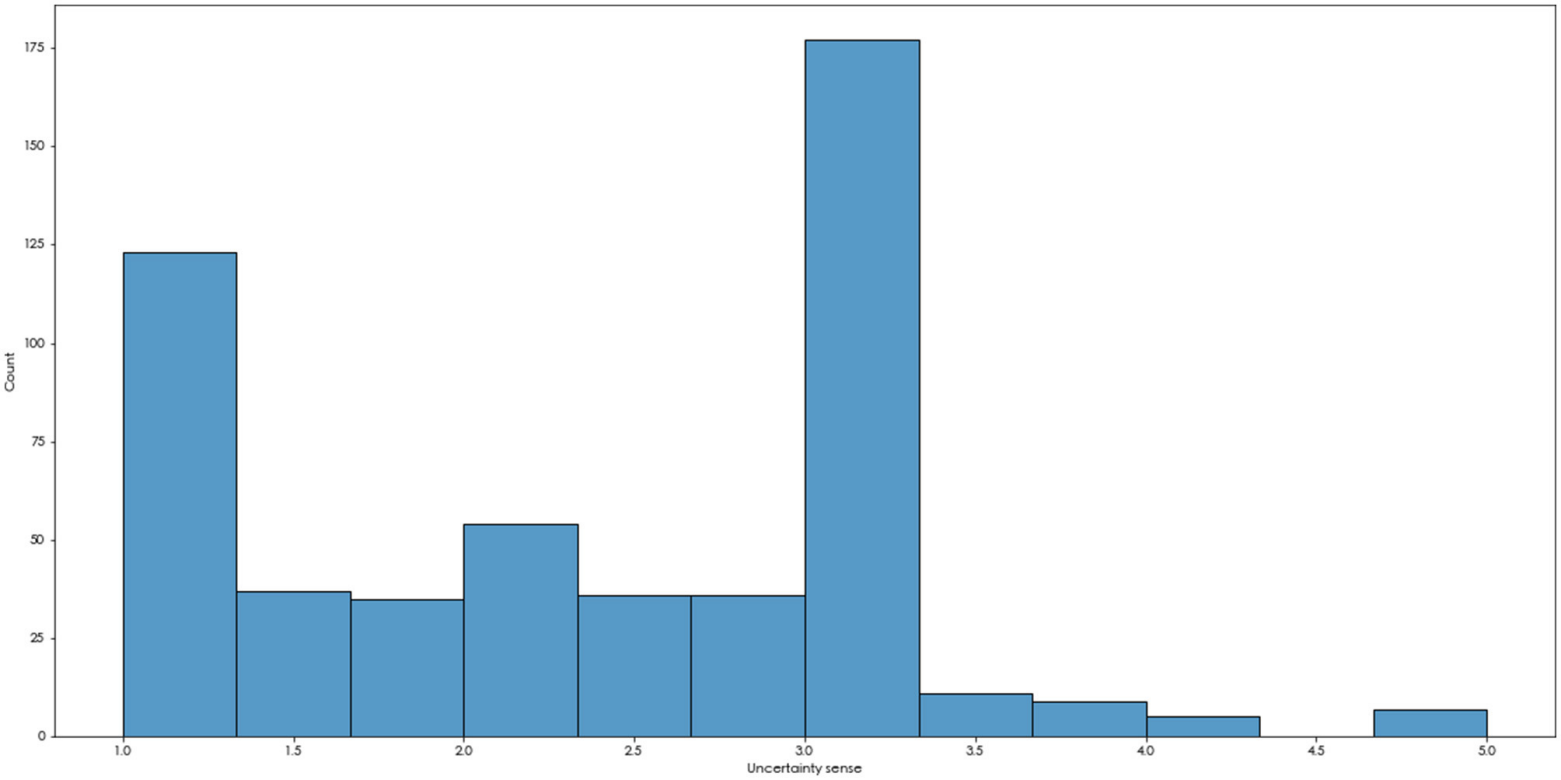
The distribution of uncertainty sense.

The scores of uncertainty ranged from 1 to 5, and 3 points indicated that the uncertainty of the subjects was in the middle level. According to the distribution of subjects' uncertainty scores (Figure 1), we divided them into two groups: the medium-high group (with scores ≥3) and the low group (with scores <3). Finally, we obtained that there were 209 subjects in the medium-high group and 311 subjects in the low group, and there were significant differences in the scores of uncertainty between such two groups [*t*_(528)_ = 29.93, *p* < 0.001].

#### 2.2.2. Depression

This study focused on depression in mental health. The Center for Epidemiologic Studies Depression Scale [CESD; (32)], was used for measuring individual levels of depression. This scale contains 20 questions, asking whether the subjects have experienced symptoms related to depression in the past few weeks. All questions were graded on the Likert scale with a 5-point scale. The higher total score of the CESD with the higher severity of depression. In this study, the Cronbach's α score for this scale was 0.90.

#### 2.2.3. Positive attitude

The self-made scale was used to measure the positive attitude of residents toward life during the pandemic. The scale contains three questions (Table 2), asking subjects about their enthusiasm to keep exercising, focus on work and cultivate interest during the pandemic. The Likert scale with 5 points was adopted for all questions (from 1 strongly disagreed to 5 strongly agreed). The higher the mean score of the subjects, the more positive the attitude toward life during the pandemic. In this study, the Cronbach's α score for this scale was 0.93.

#### 2.2.4. Preventive behavioral intentions

The self-made scale was used to measure the behavioral tendencies of residents to prevent the pandemic during the outbreak. The scale contains four questions (Table 2), asking subjects' agreement with protective behaviors such as wearing masks, hand washing and disinfection, and reducing going outside during an outbreak. The Likert scale with 5 points was adopted for all questions (from 1 strongly disagreed to 5 strongly agreed). The higher the mean score of the subjects, the higher the pandemic preventive behavior intentions. In this study, the Cronbach's α score for this scale was 0.94.

#### 2.2.5. Personality

People's inherent psychological traits can have an impact on positive life attitudes, depression levels, and pandemic preventive behavior intentions. Personality, as the sum of an individual emotions, thoughts, and behavioral tendencies, plays an important role in this process. Previous studies have shown that people's mental health (33), positive life attitudes (34) and behavioral tendencies (35) are significantly influenced by personality. For example, individuals with higher neuroticism scores were more likely to be depressed and have a pessimistic outlook on life (36, 37). Individuals with higher conscientiousness scores were more likely to adopt preventive behaviors during the pandemic (38, 39). To exclude the interference of personality on the findings, we included personality as control variables in this study. The Big Five personality scale developed by (40) was used in this study to measure the personality of the subjects. The scale contains five dimensions: neuroticism, conscientiousness, agreeableness, extraversion, and openness, and each dimension contains eight questions. The Likert scale with 5 points was adopted for all questions (from 1 very disagreed to 5 very agreed). In this study, the Cronbach's α scores were 0.91, 0.85, 0.71, 0.94, and 0.79, respectively.

#### 2.2.6. Social support

In addition to personality, the level of social support of individuals can also influence positive attitude toward life, depression levels and the preventive behavior intentions. Social support refers to the spiritual or material support given to individuals by all aspects of society, including parents, relatives, and friends (41). Relevant research shows that individuals who lack social support have more serious tendencies to depression (42) and more negative attitude toward life (43). To control for the effect of social support, we also included social support as control variables. We used the Social Support Rate Scale [SSRS; (44)] to measure the social support of the subjects. The scale contains 10 questions with three sub-dimensions: subjective support, objective support, and support utilization. In this study, the Cronbach's α scores for each dimension were 0.87, 0.77, 0.70.

#### 2.2.7. Demographic information

Besides the above scales, we also asked the participants for demographic information, including gender, geographic locations, type of household registration, health status, and the severity of the pandemic in the hometown. Geographical locations are divided according to the location of the hometown according to the following criteria: the eastern region has 13 provinces, including Beijing, Tianjin, Hebei, Shanghai, Jiangsu, Zhejiang, Fujian, Shandong, Guangdong, Hainan, Liaoning, Jilin and Heilongjiang provinces; the middle region has 6 provinces, including Shanxi, Anhui, Jiangxi, Henan, Hubei and Hunan; the western region has 12 provinces, including Inner Mongolia, Guangxi, Chongqing, Sichuan, Guizhou, Yunnan, Tibet, Shaanxi, Gansu, Qinghai, Ningxia and Xinjiang. The severity of the pandemic in the hometown was measured by a question “What do you think is the severity of the epidemic in your hometown (from not severe to very severe)”.

### 2.3. Statistical analyses

We used Python for statistical analyses. In this study, the independent variable was uncertainty sense, and the dependent variables were depression, positive life attitudes and pandemic preventive behavior intentions. Welch's analyses of variance (ANOVA) were first used to examine the relationships between demographic variables and dependent variables. Second, we performed Shapiro–Wilk test to test the normality of the data. We then performed descriptive and Pearson's correlation analyses for continuous-type variables. Variables that were significantly correlated with the dependent variable were entered into the subsequent analyses. To explore whether the effects of uncertainty differed across levels of uncertainty, we divided subjects into medium-high and low grouping based on the range and distribution of uncertainty and examined the moderating effect of group as a moderating variable. Least squares regression was conducted, and the heteroscedastic robust standard error was estimated. We used two-step regression, and the interaction terms only enter the equation of the second step. The *p*-value < 0.05 was considered to be significant.

## 3. Results

### 3.1. Impact of demographic features

We first analyzed the effects of demographic variables on pandemic preventive behavior intentions, positive life attitudes, and depression. We used Welch's ANOVA, which possesses better robustness for heteroscedasticity. The results showed in Table 3. Gender had a significant effect on depression (*F*_(1, 485)_ = 5.71, *p* = 0.017). Geography had a significant effect on depression (*F*_(2, 81)_ = 4.01, *p* = 0.022). Type of household registration had a significant effect on pandemic preventive behavior intentions (*F*_(2, 260)_ = 3.08, *p* = 0.047). Health status had a significant effect on depression (*F*_(4, 7)_ = 13.34, *p* = 0.002); Health status had a significant effect on positive attitude (*F*_(4, 7)_ = 4.73, *p* = 0.039). The severity of the pandemic in the home town had a significant effect on depression (*F*_(4, 27)_ = 14.93, *p* < 0.001); the severity of the pandemic in the home town had a significant effect on positive attitude (*F*_(4, 25)_ = 2.84, *p* = 0.046); the severity of the pandemic in the home town has a significant effect on pandemic preventive behavior intentions (*F*_(4, 27)_ = 11.77, *p* < 0.001).

**Table 3:** The effects of demographic variables on preventive behavior intentions, positive life attitudes, and depression.

		**CESD**			**Positivity**			**Behavioral intentions**		
		**M±SD**	***F***	***p***	**M±SD**	***F***	***p***	**M±SD**	***F***	***p***
Gender	Male	2.61 ± 0.75	5.71	0.017	3.66 ± 1.07	0.14	0.706	3.94 ± 1.06	0.89	0.345
	Female	2.46 ± 0.69			3.7 ± 0.93			3.86 ± 0.98		
Geography	East	2.51 ± 0.74	4.01	0.022	3.7 ± 1.02	0.84	0.84	3.94 ± 1.04	1.91	0.155
	Middle	2.72 ± 0.62			3.53 ± 0.94			3.66 ± 1.06		
	West	2.7 ± 0.7			3.7 ± 1.01			3.92 ± 0.89		
Type of household registration	City	2.56 ± 0.77	0.04	0.959	3.66 ± 1.06	1.16	0.316	3.8 ± 1.12	3.08	0.047
	Town	2.55 ± 0.72			3.56 ± 1.1			3.81 ± 1.08		
	Rural	2.54 ± 0.7			3.74 ± 0.93			4.03 ± 0.93		
Health status	Very well	2.44 ± 0.75	13.34	0.002	3.79 ± 1.03	4.73	0.039	3.95 ± 1.07	2.91	0.107
	Good	2.68 ± 0.64			3.51 ± 0.98			3.98 ± 0.93		
	General	2.96 ± 0.39			3.23 ± 0.73			3.51 ± 0.79		
	Not very well	3.16 ± 0.59			3.44 ± 1.42			3.67 ± 1.03		
	Bad	3.25 ± 0.35			3.5 ± 0.71			3.38 ± 0.53		
Severity of COVID-19	1	2.47 ± 0.75	14.93	<0.001	3.72 ± 1.05	2.84	0.046	3.97 ± 1.06	11.77	<0.001
	2	2.78 ± 0.6			3.61 ± 0.85			3.85 ± 0.89		
	3	2.86 ± 0.48			3.41 ± 0.82			3.43 ± 0.85		
	4	3.1 ± 0.21			2.94 ± 0.74			3.08 ± 0.34		
	5	2.64 ± 0.91			3.9 ± 0.79			4.35 ± 0.75		

### 3.2. Descriptive statistics and correlations

Shapiro–Wilk test show that we cannot reject the assumption of normality for all continuous variables (for all variables, *p* > 0.05). Thus Pearson' correlation analysis can be used in this study. Table 4 presents the descriptive statistics and correlation coefficients for the continuous type variables. Almost all the correlation coefficients reached a significant level (*p* <0.05), except a non-significant correlation coefficient between pandemic preventive behavior intentions and support utilization (*r* = 0.080, *p* = 0.094).

**Table 4:** Means, standard deviations, and correlation matrix of variables.

	**M±SD**	**1**	**2**	**3**	**4**	**5**	**6**	**7**	**8**	**9**	**10**	**11**	**12**
1 Neuroticism	2.39 ± 0.86	1											
2 Conscientiousness	3.64 ± 0.81	−0.317^***^	1										
3 Agreeableness	3.55 ± 0.67	−0.323^***^	0.649^***^	1									
4 Extroversion	3.55 ± 0.84	−0.109^*^	0.620^***^	0.525^***^	1								
5 Openness	3.27 ± 0.67	−0.271^***^	0.511^***^	0.406^***^	0.597^***^	1							
6 CESD	2.55 ± 0.73	0.621^***^	−0.441^***^	−0.460^***^	−0.221^***^	−0.441^***^	1						
7 Objective supports	9.11 ± 3.88	−0.188^***^	0.275^***^	0.295^***^	0.255^***^	0.303^***^	−0.290^***^	1					
8 Subjective supports	21.75 ± 5.09	−0.382^***^	0.370^***^	0.368^***^	0.248^***^	0.395^***^	−0.421^***^	0.582^***^	1				
9 Supports utilization	7.65 ± 2.31	−0.260^***^	0.232^***^	0.231^***^	0.119^**^	0.314^***^	−0.321^***^	0.303^***^	0.541^***^	1			
10 Uncertainty sense	2.25 ± 0.91	0.508^***^	−0.365^***^	−0.424^***^	−0.208^***^	−0.241^***^	0.658^***^	−0.204^***^	−0.307^***^	−0.201^***^	1		
11 Behavioral intentions	3.91 ± 1.03	−0.181^***^	0.416^***^	0.467^***^	0.412^***^	0.308^***^	−0.332^***^	0.240^***^	0.307^***^	0.080	−0.271^***^	1	
12 Positivity attitude	3.68 ± 1.01	−0.244^***^	0.465^***^	0.449^***^	0.451^***^	0.417^***^	−0.339^***^	0.248^***^	0.366^***^	0.231^***^	−0.258^***^	0.656^***^	1

^*^p <0.05, ^**^p < 0.01, ^***^p < 0.001.

### 3.3. The moderating effect of grouping variable

Taking uncertainty as the independent variable and the group variable as moderating variable, we tested whether the influence of uncertainty on depression, pandemic preventive behavior intentions, and positive attitude was regulated by the group. During the regression, variables significantly correlated with the dependent variable were entered into the equation as control variables, whereas the categorical variables were coded as dummy variables for treatment. The results are shown in Table 5. Specifically, uncertainty can significantly and positively predict CESD (β = 0.43, *p* < 0.001); whereas the group and interaction terms could not significantly predict CESD (Group: β = −0.13, *p* = 0.282; Interaction: β = 0.13, *p* = 0.241). For pandemic preventive behavior intentions, when the moderating variables were not included, the negative edge of uncertainty was significant (β = −0.08, *p* = 0.067). Whereas, when the moderating variables were included, the uncertainty had a significant positive predictive intention (β = 0.24, *p* < 0.001). Besides, group significantly and negatively predicted behavior intentions (β = −0.99, *p* < 0.001), and Interaction significantly and positively predicted behavior intentions (β = 0.60, *p* < 0.001). Simple slope analysis shows that uncertainty had no significant effect on pandemic preventive behavior intentions in low group (β = 0.002, *p* = 0.973), whereas that had a significant and positive effect (β = 0.60, *p* < 0.001) in the high group. The result of positive attitude was similar to behavioral intentions. When the moderating variables were not included, uncertainty could not significantly predict positive attitude (β = −0.02, *p* = 0.617). After the moderating variables were included, uncertainty significantly predicted positive attitude (β = 0.14, *p* = 0.047). Besides, group significantly and negatively predicted positive attitude (β = −0.55, *p* < 0.001); Interaction significantly and positively predicted positive attitude (β = 0.56, *p* < 0.001). The simple slope analysis shows that uncertainty could not significantly predict positive attitude in the low group (β = −0.08, *p* = 0.262); whereas, in the high group, uncertainty had a significant positive effect (β = 0.48, *p* < 0.001).

**Table 5:** The interaction of uncertainty sense and group on CESD, behavioral intentions and positivity.

	**CESD**				**Behavioral intentions**				**Positivity**			
	**Model 1**		**Model 2**		**Model 3**		**Model 4**		**Model 5**		**Model 6**	
	β	***SE***	β	***SE***	β	***SE***	β	***SE***	β	***SE***	β	***SE***
**Control variables**												
Neuroticism	0.293^***^	0.034	0.294^***^	0.034	0.042	0.046	0.040	0.045	−0.037	0.046	−0.033	0.045
Conscientiousness	−0.089^*^	0.041	−0.090^*^	0.042	0.049	0.057	0.057	0.055	0.107	0.056	0.103	0.055
Agreeableness	−0.107^**^	0.039	−0.108^**^	0.039	0.250^***^	0.052	0.229^***^	0.051	0.157^**^	0.052	0.150^**^	0.051
Extroversion	0.166^***^	0.040	0.161^***^	0.040	0.203^***^	0.054	0.161^**^	0.053	0.190^***^	0.053	0.166^**^	0.053
Openness	−0.245^***^	0.037	−0.244^***^	0.037	−0.005	0.050	−0.004	0.048	0.105^*^	0.049	0.111^*^	0.048
Objective support	−0.055	0.034	−0.058	0.034	0.018	0.046	0.003	0.045	−0.018	0.045	−0.029	0.045
Subjective support	−0.022	0.041	−0.024	0.041	0.112^*^	0.050	0.114^*^	0.049	0.173^***^	0.051	0.170^***^	0.053
Support utilization	−0.035	0.033	−0.034	0.033								
Female	−0.147^**^	0.056	−0.141^**^	0.057								
East	−0.052	0.104	−0.041	0.105								
Middle	−0.040	0.128	−0.026	0.129								
City					−0.232^**^	0.085	−0.229^**^	0.083				
Town					−0.207^*^	0.100	−0.173	0.096				
Healthy: very well	0.205	0.471	0.235	0.472					−0.485	0.620	−0.309	0.614
Healthy: good	0.231	0.475	0.266	0.477					−0.627	0.624	−0.431	0.619
Healthy: general	0.175	0.478	0.209	0.479					−0.502	0.628	−0.308	0.622
Healthy: not very well	0.126	0.522	0.127	0.523					−0.343	0.694	−0.305	0.686
Severity of covid-19: 1	0.296	0.202	0.324	0.204	−0.349	0.278	−0.157	0.270	−0.167	0.273	−0.044	0.271
Severity of covid-19: 2	0.234	0.215	0.268	0.217	−0.324	0.293	−0.090	0.286	−0.071	0.289	0.073	0.288
Severity of covid-19: 3	0.238	0.223	0.285	0.227	−0.584	0.303	−0.240	0.298	−0.129	0.300	0.073	0.302
Severity of covid-19: 4	0.237	0.336	0.274	0.338	−0.768	0.443	−0.574	0.430	−0.528	0.452	−0.356	0.448
**Independent variables**												
Uncertainty sense	0.389^***^	0.033	0.427^***^	0.052	−0.084	0.046	0.238^***^	0.069	−0.022	0.044	0.138^*^	0.069
Group			−0.130	0.121			−0.990^***^	0.158			−0.549^***^	0.160
**Interaction**												
Uncertainty sense × Group			0.133	0.114			0.599^***^	0.149			0.557^***^	0.150
*R^2^*	0.635		0.626		0.300		0.350		0.330		0.350	
*ΔR^2^*			0.001				0.050				0.020	

^*^p < 0.05, ^**^p < 0.01, ^***^p < 0.001.

**Figure 2:**
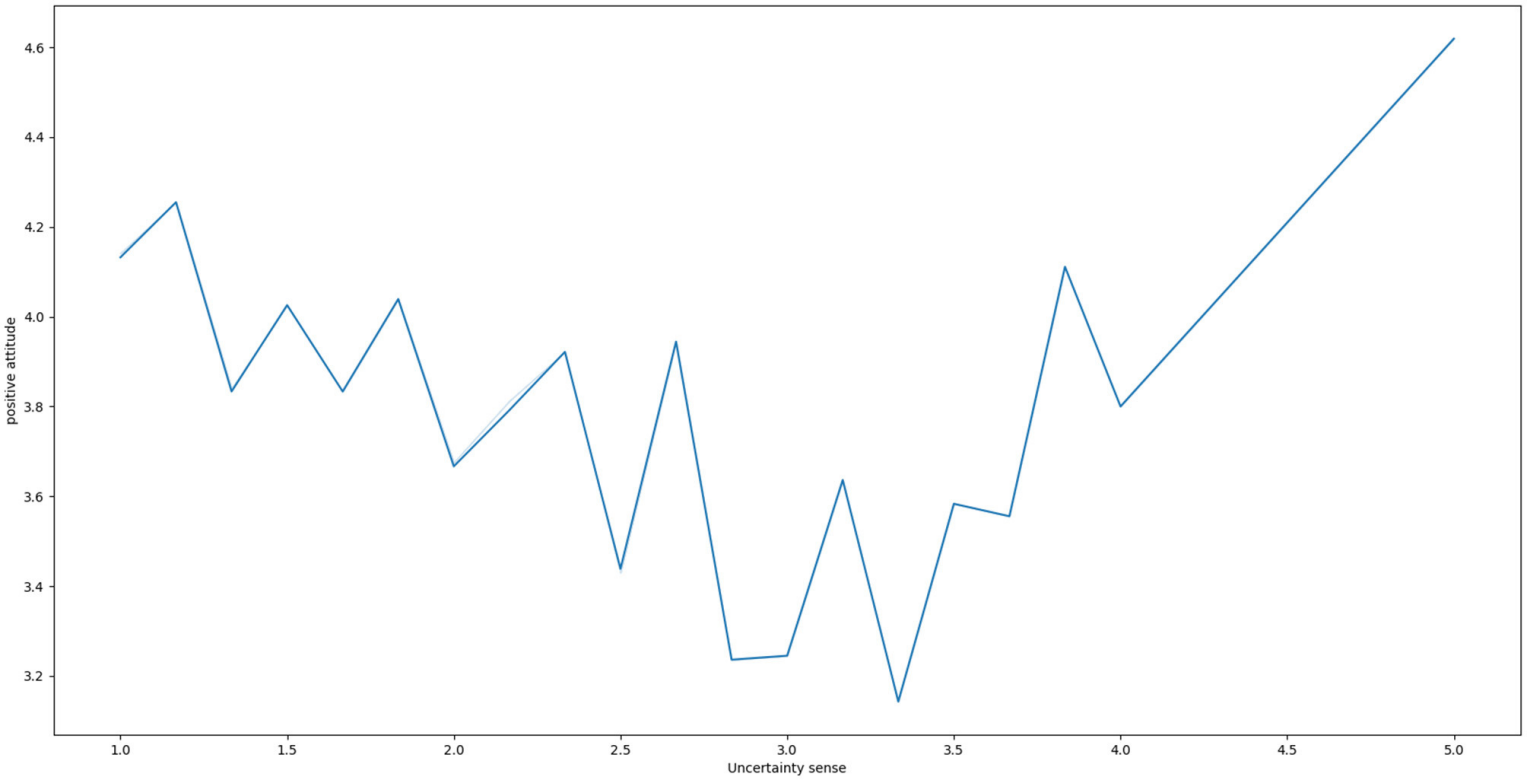
The nonlinear relationship between uncertainty sense and positive attitude.

We noted that the moderating effect of grouping was significant for positive life attitudes. Despite this, there was a significant positive effect of uncertainty on the medium-high group of uncertainty (β= 0.48, *p* < 0.001), which contradicted our hypothesis. And interestingly, the negative effect of low grouping (β = −0.08, *p* = 0.262; although not significant) seemed to imply that as uncertainty rose, the positive attitude toward life dropped firstly and then rose. Furthermore, the level of positive attitude was lowest when uncertainty was at a moderate level. The descriptive analysis confirmed our hypothesis (Figure 2). The relationship between uncertainty and positive life attitudes was “positive U-shaped”.

## 4. Discussion

This study proposes a sense of uncertainty to better describe people's perceptions of uncertainty about the future development of the pandemic and uncertainty about the spread of the pandemic and their own being infected at a time when the pandemic is gradually being controlled. And based on this, this study explores the effects of this uncertainty on people's positive life attitudes, depression levels, and pandemic preventive behavior intentions. This study further shows that levels of uncertainty can moderate the influence of uncertainty on mental states. Specifically, when the level of pandemic uncertainty is high, people's pandemic preventive behavior intentions and depression levels increase and positive life attitudes diminish as uncertainty increases; whereas, when the uncertainty level is low, this influence will be reduced or even disappear. Using an online questionnaire, this study collected data on uncertainty, positive life attitudes, depression, pandemic preventive behavior intentions, personality, and social support from 530 subjects, and tested the hypothesis through an analysis of moderate effects.

The results of the analysis partially supported our hypothesis. The group variable did not have a significant moderating effect on depression levels. This suggested that an increase in uncertainty consistently resulted in an increase in depression levels, regardless of whether the individual was at a high or low level of uncertainty. This monotonic linear relationship was consistent with the evidence from sense of control and uncertainty tolerance (9, 12, 14, 18, 26). For positive life attitudes, although the moderating effect of group was significant, the effect was in the opposite direction of our hypothesis for the medium-high group. Our further analysis also revealed that the relationship between uncertainty and positive life attitudes was “positive U-shaped”. Specifically, at low uncertainty, positive life attitudes decreased with increasing uncertainty (although not significantly), whereas at high uncertainty, positive life attitudes increased with increasing uncertainty. To our knowledge, this is the first time since the pandemic that uncertainty (or its associated sense of control and uncertainty tolerance) has been found to have a nonlinear effect on people's psychological states.

This phenomenon may be related to the psychological typhoon eye effect (45). A psychological typhoon eye refers to the phenomenon that individuals in the central area where a disaster occurs have a calmer psychological reaction than those outside the central area. The difference between the COVID-19 pandemic and other disasters (such as earthquakes) is that there is no fixed disaster center. When individuals feel a strong sense of uncertainty, they perceive the risk of the spread of the pandemic and the risk of being infected themselves to be high. At this time, although individuals are not spatially at the center of the outbreak, they are psychologically closer to the center of the disaster. According to the mere exposure effect (46), this group of individuals has been exposed to high levels of uncertainty for a long time, and over time has developed adaptability to tolerate the great uncertainty and face daily life with a positive attitude. We believe that this nonlinear relationship may be a newly emerged phenomenon in the post-pandemic era. On one hand, the current pandemic is gradually under control. People's uncertainty mainly comes from subjective feelings rather than the huge real threat at the beginning of the outbreak. On the other hand, it takes some time for people to develop adaptability.

These results have given us some insights. First, it's necessary to pay extra attention to those with moderate levels of uncertainty. The depression level of this part of the population is at the medium level, but the life state under the pandemic situation is the most negative. This group is more likely to have serious mental health problems. Although people with higher levels of uncertainty have higher levels of depression, they can face life with a more positive attitude due to adaptability—they may have a stronger tolerance. Second, it may be meaningful to explore the nonlinear relationship between psychological traits. We found an interesting phenomenon during the analysis: the prediction of uncertainty on positive attitude and pandemic preventive behavior intentions was insignificant when group variables and interaction terms were not included, whereas, after inclusion, this effect reached significance levels. This shows that when analyzing as a whole, the influence of each sub-sample is ignored. At the beginning of the pandemic outbreak, people's collective fear and uncertainty entered a higher level. At this point, the samples are relatively homogeneous, and the conclusions obtained from the research are specific to the high group. And it remains uncertain whether these findings can be replicated across groups. Third, it is necessary to define psychological traits according to the different phases of the pandemic and to explore the relationship between psychological traits as they change with the pandemic. It is important to note that concepts such as sense of control and uncertainty, which were widely studied at the beginning of the outbreak, may not be applicable to the current phase of the pandemic. However, defining more relevant psychological characteristics will help us to accurately expose the relationship between the pandemic and people's psychological state. Moreover, we need to pay attention to the changes of this relationship with the development of the pandemic. As mentioned before, we assume that the nonlinear relationship between uncertainty and positive attitude toward life may just appear at the moment. This needs to be verified by further research. On the other hand, it also inspires us to explore whether the relationship between the psychological traits confirmed at the initial stage of the pandemic has changed in the post-pandemic era.

There are some limitations in this research. First, this paper collects data and conducts research in China. Unlike other countries, China has implemented epidemic control measures for a long time, which may lead to the specificity of the results found in China. Thus for future research, we propose to examine the generalizability of the findings through cross-cultural studies and controlling for the objective severity of the pandemic. Second, this study mainly focused on college students in Dalian city. For the follow-up research, we plan to make use of big data from social media (Weibo) to see if the conclusions can be reproduced, since social media can provide a larger amount of data at a lower cost and helps us to test the robustness of our conclusions in large samples.

## 5. Conclusion

To explore the nonlinear effects of pandemic uncertainty on depression, pandemic preventive behavior intentions, and positive attitude, this paper first grouped uncertainty high and low and conducted a moderating effect analysis using the group variable as a moderating variable. This study found that the group variable did not significantly regulate the influence of uncertainty on depression, but significantly regulated the influence of uncertainty on positive attitude and pandemic preventive behavior intentions. Further simple slope analyses found that the high group significantly and positively predicted positive attitude and pandemic preventive behavior intentions, while the low group effects were not significant. This research found a nonlinear influence of pandemic uncertainty on people's psychological characteristics, which was rare in the early stages of a pandemic outbreak. This reveals the need to explore the non-linear relationship of psychological traits under the pandemic, and to observe the relationship of psychological traits with the pandemic.

## Data availability statement

The raw data supporting the conclusions of this article will be made available by the authors, without undue reservation.

## Ethics statement

The studies involving human participants were reviewed and approved by the Institutional Review Board of the Institute of Psychology, Chinese Academy of Sciences (H16003). The patients/participants provided their written informed consent to participate in this study.

## Author contributions

TZ provided financial support for the study. TZ and AL conceived and planned the article. YL and ZL carried out the study and drafted the manuscript. ZL developed the tools needed for the experiment, executed the whole experiment process, performed all of the statistical analyses, and wrote the manuscript with input from all authors. YL made a great contribution to the revision of the manuscript. All authors contributed to the article and approved the submitted version.
